# Supplementary material for: CAR-T Cell Therapy for Acute Myeloid Leukemia: Where Do We Stand Now?
Source: Curr Oncol. 2025 May 30;32(6):322. doi: 10.3390/curroncol32060322 (PMC12191959; doi:10.3390/curroncol32060322)
Supplement: Supplementary file 1 [file curroncol-32-00322-s001.zip › curroncol-3654519-supplementary.pdf]

Supplementary Material

CAR-T Cell Therapy for Acute Myeloid Leukemia: Where do We Stand Now?

Pilar Lloret-Madrid [1,2], Pedro Chorão [1,2], Manuel Guerreiro [1,2] and Pau Montesinos [1,3]*

[1] Hematology Department, Hospital Universitari i Politècnic La Fe, Valencia, Spain
[2] Instituto de Investigación Sanitaria La Fe (IISLAFE), Valencia, Spain
[3] Department of Medicine, University of Valencia, Spain
* Correspondence: Pau Montesinos, montesinos_pau@gva.es

Supplementary Table S1. PRISMA 2020 Checklist.

Section and Topic	Item #	Checklist item	Location where item is reported (page)
**TITLE**			
Title	1	Identify the report as a systematic review.	1
**ABSTRACT**			
Abstract	2	See the PRISMA 2020 for Abstracts checklist.	Not performed
**INTRODUCTION**			
Rationale	3	Describe the rationale for the review in the context of existing knowledge.	1-2
Objectives	4	Provide an explicit statement of the objective(s) or question(s) the review addresses.	2
**METHODS**			
Eligibility criteria	5	Specify the inclusion and exclusion criteria for the review and how studies were grouped for the syntheses.	2
Information sources	6	Specify all databases, registers, websites, organisations, reference lists and other sources searched or consulted to identify studies. Specify the date when each source was last searched or consulted.	2
Search strategy	7	Present the full search strategies for all databases, registers and websites, including any filters and limits used.	2
Selection process	8	Specify the methods used to decide whether a study met the inclusion criteria of the review, including how many reviewers screened each record and each report retrieved, whether they worked independently, and if applicable, details of automation tools used in the process.	2
Data collection process	9	Specify the methods used to collect data from reports, including how many reviewers collected data from each report, whether they worked independently, any processes for obtaining or confirming data from study investigators, and if applicable, details of automation tools used in the process.	2
Data items	10a	List and define all outcomes for which data were sought. Specify whether all results that were compatible with each outcome domain in each study were sought (e.g. for all measures, time points, analyses), and if not, the methods used to decide which results to collect.	3
	10b	List and define all other variables for which data were sought (e.g. participant and intervention characteristics, funding sources). Describe any assumptions made about any missing or unclear information.	3
Study risk of bias assessment	11	Specify the methods used to assess risk of bias in the included studies, including details of the tool(s) used, how many reviewers assessed each study and whether they worked independently, and if applicable, details of automation tools used in the process.	3
Effect measures	12	Specify for each outcome the effect measure(s) (e.g. risk ratio, mean difference) used in the synthesis or presentation of results.	Not

Section and Topic	Item #	Checklist item	Location where item is reported (page)
			performed
Synthesis methods	13a	Describe the processes used to decide which studies were eligible for each synthesis (e.g. tabulating the study intervention characteristics and comparing against the planned groups for each synthesis (item #5)).	Not performed
	13b	Describe any methods required to prepare the data for presentation or synthesis, such as handling of missing summary statistics, or data conversions.	Not performed
	13c	Describe any methods used to tabulate or visually display results of individual studies and syntheses.	Not performed
	13d	Describe any methods used to synthesize results and provide a rationale for the choice(s). If meta-analysis was performed, describe the model(s), method(s) to identify the presence and extent of statistical heterogeneity, and software package(s) used.	Not performed
	13e	Describe any methods used to explore possible causes of heterogeneity among study results (e.g. subgroup analysis, meta-regression).	Not performed
	13f	Describe any sensitivity analyses conducted to assess robustness of the synthesized results.	Not performed
Reporting bias assessment	14	Describe any methods used to assess risk of bias due to missing results in a synthesis (arising from reporting biases).	Not performed
Certainty assessment	15	Describe any methods used to assess certainty (or confidence) in the body of evidence for an outcome.	3
**RESULTS**			
Study selection	16a	Describe the results of the search and selection process, from the number of records identified in the search to the number of studies included in the review, ideally using a flow diagram.	3
	16b	Cite studies that might appear to meet the inclusion criteria, but which were excluded, and explain why they were excluded.	3
Study characteristics	17	Cite each included study and present its characteristics.	6-10
Risk of bias in studies	18	Present assessments of risk of bias for each included study.	3
Results of individual studies	19	For all outcomes, present, for each study: (a) summary statistics for each group (where appropriate) and (b) an effect estimate and its precision (e.g. confidence/credible interval), ideally using structured tables or plots.	Not performed
Results of	20a	For each synthesis, briefly summarise the characteristics and risk of bias among contributing studies.	3, 6-8

Section and Topic	Item #	Checklist item	Location where item is reported (page)
syntheses	20b	Present results of all statistical syntheses conducted. If meta-analysis was done, present for each the summary estimate and its precision (e.g. confidence/credible interval) and measures of statistical heterogeneity. If comparing groups, describe the direction of the effect.	Not performed
	20c	Present results of all investigations of possible causes of heterogeneity among study results.	Not performed
	20d	Present results of all sensitivity analyses conducted to assess the robustness of the synthesized results.	Not performed
Reporting biases	21	Present assessments of risk of bias due to missing results (arising from reporting biases) for each synthesis assessed.	Not performed
Certainty of evidence	22	Present assessments of certainty (or confidence) in the body of evidence for each outcome assessed.	Not performed
**DISCUSSION**			
Discussion	23a	Provide a general interpretation of the results in the context of other evidence.	10-11
	23b	Discuss any limitations of the evidence included in the review.	10-11
	23c	Discuss any limitations of the review processes used.	Not performed
	23d	Discuss implications of the results for practice, policy, and future research.	10-15
**OTHER INFORMATION**			
Registration and protocol	24a	Provide registration information for the review, including register name and registration number, or state that the review was not registered.	2
	24b	Indicate where the review protocol can be accessed, or state that a protocol was not prepared.	Protocol was not prepared
	24c	Describe and explain any amendments to information provided at registration or in the protocol.	Not applicable
Support	25	Describe sources of financial or non-financial support for the review, and the role of the funders or sponsors in the review.	Non-financial support
Competing interests	26	Declare any competing interests of review authors.	No competing

Section and Topic	Item #	Checklist item	Location where item is reported (page)
			interests
Availability of data, code and other materials	27	Report which of the following are publicly available and where they can be found: template data collection forms; data extracted from included studies; data used for all analyses; analytic code; any other materials used in the review.	Not applicable

From: Page MJ, McKenzie JE, Bossuyt PM, Boutron I, Hoffmann TC, Mulrow CD, et al. The PRISMA 2020 statement: an updated guideline for reporting systematic reviews. BMJ 2021;372:n71. doi: 10.1136/bmj.n71. This work is licensed under CC BY 4.0. To view a copy of this license, visit https://creativecommons.org/licenses/by/4.0/

Supplementary Table S2. Summary of study quality and risk of bias.

Target	Phase	N	Publication type	Follow-up	Outcome reporting	Safety reporting	Risk of bias	Comments	Ref
**CD7**	I/II	1	Correspondence	2 months	Partial	Partial	High	Single case	[1]
	I	12	Original article	Median 10.5 months (95% CI 10.2 – 10.8)	Partial	Complete	High - moderate	Single-arm, non-randomized, small sample size	[2]
	I	9	Abstract (conference)	Median 5.4 months (range 1.0 – 9.8)	Partial	Partial	High	Single-arm, non-randomized, small sample size	[3]
**CD19**	II	6	Original article	2 – 15 months	Complete	Complete	High - moderate	Single-arm, non-randomized, small sample size	[4]
	N/A	8	Letter	10 – 26 months	Partial	Partial	High	Single-arm, non-randomized, small sample size	[5]
**CD33**	I	10	Letter	Not reported	Partial	Complete	High	Single-arm, non-randomized, small sample size	[6]
	I	1	Original article	Not reported	Complete	Complete	High	Single case	[7]
	I/Ib	24	Abstract (conference)	Not reported	Partial	Partial	High	Single-arm, non-randomized, small sample size	[8]
	I/II	24	Abstract (conference)	Not reported	Partial	Partial	High	Single-arm, non-randomized, small sample size	[9]
	I/II	4	Original article	Until 26 months	Complete	Partial	High	Single-arm, non-randomized, small sample size	[10]
	N/A	12	Abstract (conference)	4 – 12 months	Partial	Partial	High	Single-arm, non-randomized, small sample size	[11]
**CD38**	I/II	6	Letter	Until 9 months	Complete	Complete	High - moderate	Single-arm, non-randomized, small sample size	[12]
**CD123**	I	19	Abstract (conference)	Until 5 months	Partial	Partial	High	Single-arm, non-randomized, small sample size	[13]
	I	16	Abstract (conference)	Until 8 months	Partial	Partial	High	Single-arm, non-randomized, small sample size	[14]
	I	14	Abstract (conference)	1 – 5 months	Partial	Partial	High	Single-arm, non-randomized, small sample size	[15]

	I	12	Abstract (conference)	1.5 – 2 months	Partial	Partial	High	Single-arm, non-randomized, small sample size	[16]
	N/A	1	Case report	~ 2 months	Complete	Complete	Hight	Single case	[17]
**CLL-1**	I/II	8	Original article	Not reported	Complete	Partial	High	Single-arm, non-randomized, small sample size	[18]
	I	47	Original article	Median 9 months (range 2 – 30)	Complete	Complete	Moderate	Single-arm, non-randomized	[19]
	I/II	2	Original article	3 – 8 months	Partial	Partial	High	Single-arm, non-randomized, small sample size	[20]
	N/A	1	Case report	Until 4 months	Partial	Partial	High	Single case	[21]
**CLL-1-CD33**	I	9	Abstract (conference)	Not reported	Partial	Partial	High	Single-arm, non-randomized, small sample size	[22]
**NKG2D-L**	I	25	Original article	Median 118 days (IQR 46 – 180)	Partial	Complete	High - moderate	Single-arm, non-randomized, small sample size	[23]
	I	14	Original article	Until 26 months	Complete	Complete	High - moderate	Single-arm, non-randomized, small sample size	[24]
	I	11	Abstract (conference)	Not reported	Partial	Partial	High	Single-arm, non-randomized, small sample size	[25]

CI: confidence interval; IQR: interquartile range.

References

1. Cao, X.; Dai, H.; Cui, Q.; Li, Z.; Shen, W.; Pan, J.; Shen, H.; Ma, Q.; Li, M.; Chen, S.; et al. CD7-Directed CAR T-Cell Therapy: A Potential Immunotherapy Strategy for Relapsed/Refractory Acute Myeloid Leukemia. *Exp Hematol Oncol* **2022**, *11*, 67, doi:10.1186/s40164-022-00318-6.
2. Hu, Y.; Zhou, Y.; Zhang, M.; Zhao, H.; Wei, G.; Ge, W.; Cui, Q.; Mu, Q.; Chen, G.; Han, L.; et al. Genetically Modified CD7-Targeting Allogeneic CAR-T Cell Therapy with Enhanced Efficacy for Relapsed/Refractory CD7-Positive Hematological Malignancies: A Phase I Clinical Study. *Cell Res* **2022**, *32*, 995–1007, doi:10.1038/s41422-022-00721-y.
3. Hu Y, Zhang M, Zhao H, et al. High Safety and Efficacy of Anti-CD7 CAR-T Cells in Treating Relapsed or Refractory CD7+ Acute Myeloid Leukemia: First-in-Human Phase I Study. In: 49th EBMT Annual Meeting, Paris, France, 23–26 April 2023.
4. Danylesko, I.; Shem-Tov, N.; Yerushalmi, R.; Jacoby, E.; Toren, A.; Shouval, R.; Itzhaki, O.; Avigdor, A.; Shimoni, A.; Nagler, A. Point of Care CD19 Chimeric Antigen Receptor (CAR) T-Cells for Relapsed/Refractory Acute Myeloid Leukemia (AML) with Aberrant CD19 Antigen Expression. *Curr Res Transl Med* **2024**, *72*, 103471, doi:10.1016/j.retram.2024.103471.
5. Liu, S.; Yin, Z.; Yu, X.; Zhao, Y.; Pan, J.; Song, Y. CD19-Specific CAR-T Cell Therapy for Relapsed/Refractory Non-B-Cell Acute Leukaemia with CD19 Antigen Expression. *European Journal of Cancer* **2021**, *153*, 1–4, doi:10.1016/j.ejca.2021.04.042.
6. Tambaro, F.P.; Singh, H.; Jones, E.; Rytting, M.; Mahadeo, K.M.; Thompson, P.; Daver, N.; DiNardo, C.; Kadia, T.; Garcia-Manero, G.; et al. Autologous CD33-CAR-T Cells for Treatment of Relapsed/Refractory Acute Myelogenous Leukemia. *Leukemia* **2021**, *35*, 3282–3286, doi:10.1038/s41375-021-01232-2.
7. Wang, Q.; Wang, Y.; Lv, H.; Han, Q.; Fan, H.; Guo, B.; Wang, L.; Han, W. Treatment of CD33-Directed Chimeric Antigen Receptor-Modified T Cells in One Patient With Relapsed and Refractory Acute Myeloid Leukemia. *Molecular Therapy* **2015**, *23*, 184–191, doi:10.1038/mt.2014.164.
8. Sallman, D.A.; Elmariah, H.; Sweet, K.; Mishra, A.; Cox, C.A.; Chakaith, M.; Semnani, R.; Shehzad, S.; Anderson, A.; Sabzevari, H.; et al. Phase 1/1b Safety Study of Prgn-3006 Ultracar-T in Patients with Relapsed or Refractory CD33-Positive Acute Myeloid Leukemia and Higher Risk Myelodysplastic Syndromes. *Blood* **2022**, *140*, 10313–10315, doi:10.1182/blood-2022-169142.
9. Shah, N.N.; Tasian, S.K.; Kohler, M.E.; Hsieh, E.M.; Baumeister, S.H.C.; Summers, C.; Shalabi, H.; Pollard, J.A.; Yates, B.; Brazauskas, R.; et al. CD33 CAR T-Cells (CD33CART) for Children and Young Adults with Relapsed/Refractory AML: Dose-Escalation Results from a Phase I/II Multicenter Trial. *Blood* **2023**, *142*, 771–771, doi:10.1182/blood-2023-179667.
10. Zuo, S.; Li, C.; Sun, X.; Deng, B.; Zhang, Y.; Han, Y.; Ling, Z.; Xu, J.; Duan, J.; Wang, Z.; et al. C-JUN Overexpressing CAR-T Cells in Acute Myeloid Leukemia: Preclinical Characterization and Phase I Trial. *Nat Commun* **2024**, *15*, 6155, doi:10.1038/s41467-024-50485-9.
11. Lin, Y.; Zhao, D.; Deng, B.; Liu, D.; Yan, H.; Li, B.; Xia, Y.; Zheng, R.; Wu, T.; Tong, C. The Safety and Efficacy of CD33 CAR-T Therapy for RR AML after HSCT. *Blood* **2024**, *144*, 3467–3467, doi:10.1182/blood-2024-207659.
12. Cui, Q.; Qian, C.; Xu, N.; Kang, L.; Dai, H.; Cui, W.; Song, B.; Yin, J.; Li, Z.; Zhu, X.; et al. CD38-Directed CAR-T Cell Therapy: A Novel Immunotherapy Strategy for Relapsed Acute Myeloid Leukemia after Allogeneic Hematopoietic Stem Cell Transplantation. *J Hematol Oncol* **2021**, *14*, 82, doi:10.1186/s13045-021-01092-4.
13. Wermke, M.; Metzelder, S.; Kraus, S.; Sala, E.; Vucinic, V.; Fiedler, W.; Wetzko, K.; Schäfer, J.; Goebeler, M.-E.; Koedam, J.; et al. Updated Results from a Phase I Dose Escalation Study of the Rapidly-Switchable Universal CAR-T Therapy UniCAR-T-CD123 in Relapsed/Refractory AML. *Blood* **2023**, *142*, 3465–3465, doi:10.1182/blood-2023-177867.

14. Sallman, D.A.; DeAngelo, D.J.; Pemmaraju, N.; Dinner, S.; Gill, S.; Olin, R.L.; Wang, E.S.; Konopleva, M.; Stark, E.; Korngold, A.; et al. Ameli-01: A Phase I Trial of UCART123v1.2, an Anti-CD123 Allogeneic CAR-T Cell Product, in Adult Patients with Relapsed or Refractory (R/R) CD123+ Acute Myeloid Leukemia (AML). *Blood* **2022**, *140*, 2371–2373, doi:10.1182/blood-2022-169928.
15. Budde, L.; Song, J.Y.; Kim, Y.; Blanchard, S.; Wagner, J.; Stein, A.S.; Weng, L.; Del Real, M.; Hernandez, R.; Marcucci, E.; et al. Remissions of Acute Myeloid Leukemia and Blastic Plasmacytoid Dendritic Cell Neoplasm Following Treatment with CD123-Specific CAR T Cells: A First-in-Human Clinical Trial. *Blood* **2017**, *130*, 811–811, doi:10.1182/blood.V130.Suppl_1.811.811.
16. Naik, S.; Madden, R.M.; Lipsitt, A.; Lockey, T.; Bran, J.; Rubnitz, J.E.; Klco, J.; Shulkin, B.; Patil, S.L.; Schell, S.; et al. Safety and Anti-Leukemic Activity of CD123-CAR T Cells in Pediatric Patients with AML: Preliminary Results from a Phase 1 Trial. *Blood* **2022**, *140*, 4584–4585, doi:10.1182/blood-2022-170201.
17. Yao, S.; Jianlin, C.; Yarong, L.; Botao, L.; Qinghan, W.; Hongliang, F.; Lu, Z.; Hongmei, N.; Pin, W.; Hu, C.; et al. Donor-Derived CD123-Targeted CAR T Cell Serves as a RIC Regimen for Haploidentical Transplantation in a Patient With FUS-ERG+ AML. *Front. Oncol.* **2019**, *9*, 1358, doi:10.3389/fonc.2019.01358.
18. Zhang, H.; Bu, C.; Peng, Z.; Li, G.; Zhou, Z.; Ding, W.; Zheng, Y.; He, Y.; Hu, Z.; Pei, K.; et al. Characteristics of Anti-CLL1 Based CAR-T Therapy for Children with Relapsed or Refractory Acute Myeloid Leukemia: The Multi-Center Efficacy and Safety Interim Analysis. *Leukemia* **2022**, *36*, 2596–2604, doi:10.1038/s41375-022-01703-0.
19. Zhao, Y.; Bai, X.; Guo, S.; Zhang, X.; Liu, J.; Zhao, M.; Xie, T.; Meng, H.; Zhang, Y.; He, X.; et al. Efficacy and Safety of CAR-T Therapy Targeting CLL1 in Patients with Extramedullary Diseases of Acute Myeloid Leukemia. *J Transl Med* **2024**, *22*, 888, doi:10.1186/s12967-024-05705-7.
20. Ma, Y.-J.; Dai, H.-P.; Cui, Q.-Y.; Cui, W.; Zhu, W.-J.; Qu, C.-J.; Kang, L.-Q.; Zhu, M.-Q.; Zhu, X.-M.; Liu, D.-D.; et al. Successful Application of PD-1 Knockdown CLL-1 CAR-T Therapy in Two AML Patients with Post-Transplant Relapse and Failure of Anti-CD38 CAR-T Cell Treatment.
21. Miao, X.; Shuai, Y.; Han, Y.; Zhang, N.; Liu, Y.; Yao, H.; Wang, X.; He, G.; Chen, D.; Fan, F.; et al. Case Report: Donor-Derived CLL-1 Chimeric Antigen Receptor T-Cell Therapy for Relapsed/Refractory Acute Myeloid Leukemia Bridging to Allogeneic Hematopoietic Stem Cell Transplantation after Remission. *Front Immunol* **2024**, *15*, 1389227, doi:10.3389/fimmu.2024.1389227.
22. Fang Liu, Hongyu Zhang, Lihua Sun, Yecheng Li, Shan Zhang, Guangcui He, Hai Yi, Masayuki Wada, Kevin G Pinz, Kevin H Chen, Yu Ma, Yisong Xiong, Yi Su, Yupo Ma FIRST-IN-HUMAN CLL1-CD33 COMPOUND CAR (CCAR) T CELL THERAPY IN RELAPSED AND REFRACTORY ACUTE MYELOID LEUKEMIA. *25th EHA Annual Congress. 2020, 12: Frankfurt, Germany. EHA Library. Liu F. 06/12/2020; 294969; S149*.
23. Sallman, D.A.; Kerre, T.; Havelange, V.; Poiré, X.; Lewalle, P.; Wang, E.S.; Brayer, J.B.; Davila, M.L.; Moors, I.; Machiels, J.-P.; et al. CYAD-01, an Autologous NKG2D-Based CAR T-Cell Therapy, in Relapsed or Refractory Acute Myeloid Leukaemia and Myelodysplastic Syndromes or Multiple Myeloma (THINK): Haematological Cohorts of the Dose Escalation Segment of a Phase 1 Trial. *The Lancet Haematology* **2023**, *10*, e191–e202, doi:10.1016/S2352-3026(22)00378-7.
24. Baumeister, S.H.; Murad, J.; Werner, L.; Daley, H.; Trebeden-Negre, H.; Gicobi, J.K.; Schmucker, A.; Reder, J.; Sentman, C.L.; Gilham, D.E.; et al. Phase I Trial of Autologous CAR T Cells Targeting NKG2D Ligands in Patients with AML/MDS and Multiple Myeloma. *Cancer Immunology Research* **2019**, *7*, 100–112, doi:10.1158/2326-6066.CIR-18-0307.
25. Deeren, D.; Maertens, J.A.; Lin, T.; Beguin, Y.; Demoulin, B.; Fontaine, M.; Sotiropoulou, P.A.; Alcantar-Orozco, E.; Breman, E.; Dheur, M.-S.; et al. First Results from the Dose Escalation Segment of the Phase I Clinical Study Evaluating Cyad-02, an Optimized Non Gene-Edited Engineered NKG2D CAR T-Cell Product, in Relapsed or Refractory Acute Myeloid Leukemia and Myelodysplastic Syndrome Patients. *Blood* **2020**, *136*, 36–36, doi:10.1182/blood-2020-139667.
